# All‐Optical Modulation with Dielectric Nanoantennas: Multiresonant Control and Ultrafast Spatial Inhomogeneities

**DOI:** 10.1002/smsc.202000079

**Published:** 2021-05-07

**Authors:** Andrea Mazzanti, Eva Arianna Aurelia Pogna, Lavinia Ghirardini, Michele Celebrano, Andrea Schirato, Giuseppe Marino, Aristide Lemaítre, Marco Finazzi, Costantino De Angelis, Giuseppe Leo, Giulio Cerullo, Giuseppe Della Valle

**Affiliations:** ^1^ Dipartimento di Fisica Politecnico di Milano Piazza Leonardo da Vinci 32 20133 Milano Italy; ^2^ NEST Istituto Nanoscienze-CNR and Scuola Normale Superiore 56127 Pisa Italy; ^3^ Istituto Italiano di Tecnologia via Morego 30 I-16163 Genova Italy; ^4^ Matériaux et Phénomènes Quantiques Universitè de Paris-CNRS F-75013 Paris France; ^5^ Centre de Nanosciences et de Nanotechnologies CNRS & Université Paris-Saclay Palaiseau France; ^6^ Dipartimento di Ingegneria dell'Informazione Université di Brescia Via Branze 38 I-25123 Brescia Italy; ^7^ Istituto di Fotonica e Nanotecnologie Consiglio Nazionale delle Ricerche Piazza Leonardo da Vinci 32 20133 Milano Italy

**Keywords:** AlGaAs, all-dielectric nanoantennas, nonlinear nanophotonics, pump-probe spectroscopy, ultrafast nanophotonics

## Abstract

The transient optical response of multiresonant all‐dielectric nanoantennas via a combination of broadband ultrafast reflectivity experiments and nonlinear optics nanoscale modeling is studied. Ultrafast all‐optical control of the reflectivity is demonstrated in variably sized ${\text{Al}}_{.18}{\text{Ga}}_{.82}\text{As}$ nanoantennas over four distinct Mie resonances (including Fano‐like resonances), spanning a broad spectral range, from the red to the near‐infrared. A spatially inhomogeneous dynamical model, which accounts for diffusion of the photogenerated carriers inside the semiconductor, is introduced and exploited to isolate the physical phenomena leading to the overall transient response, namely, Drude plasma formation and Pauli blocking following band filling and thermo‐optical effect. The results pave the way to the development of multiwavelength all‐optically reconfigurable filters for next‐generation ultrafast add/drop multiplexing.

## Graphical abstract

All‐dielectric nanoantennas enable to control light fields via excitation of Mie resonances inducing ultrafast all‐optical reflectivity switching. Herein, combining inhomogeneous modeling of the photoexcited charge‐carrier dynamics and ultrafast pump‐probe spectroscopy experiments, the capability of simultaneous modulation of multiple geometrically tunable resonances in the visible and near‐infrared ranges is demonstrated.

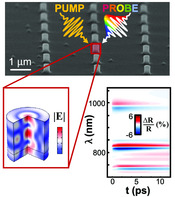

## 1 Introduction

Optical nanoantennas are subwavelength resonant nanostructures based on high permittivity media, being either metallic or all‐dielectric.^[^ ^1^, ^2^, ^3^ ^]^ Similar to macroscopic antennas, their resonant response can be designed by acting on the geometrical parameters, making it possible to achieve refined control over the scattered optical fields. Especially in 2D arrangements (also known as metasurfaces), optical nanoantennas have disclosed unprecedented possibilities for the molding of light in ultracompact configurations.^[^ ^4^, ^5^, ^6^, ^7^, ^8^ ^]^ Most recently, they have also been proposed for nonlinear applications, opening a new frontier of research now referred to as nonlinear nanophotonics.^[^ ^9^, ^10^ ^]^

The key factors that enable strong nonlinear optical effects at the nanoscale are a high field enhancement inside the nanostructures and a high nonlinear susceptibility of the constituent medium. This, at first, led to the choice of noble metals for the design of nonlinear nanomaterials,^[^ ^11^, ^12^, ^13^ ^]^ due to their giant third‐order nonlinearity^[^ ^14^, ^15^ ^]^ and high field enhancements provided by plasmonic resonances. However, noble metals suffer from strong energy dissipation due to Ohmic losses.^[^ ^16^ ^]^ For this reason, new material platforms have been the subject of intensive research.

All‐dielectric nanoantennas have been proposed as alternatives to metallic ones^[^ ^17^ ^]^ because of the possibility to excite Mie resonances inside nanostructures made of high refractive index materials experiencing much lower Ohmic losses.^[^ ^18^ ^]^ Promising candidates for such configurations, also in view of their high third‐order optical nonlinearity, are conventional semiconductors, including silicon,^[^ ^19^, ^20^ ^]^ that can be easily operated in transmission when the nanoantennas are supported on a silica substrate, and gallium arsenide^[^ ^21^ ^]^ and gallium phosphide,^[^ ^22^ ^]^ mostly suitable for high reflectivity configurations. In particular, all‐optical reflectivity modulation on a few picoseconds time scale has been demonstrated from GaAs nanoantennas^[^ ^21^ ^]^ by exploiting the magnetic dipole resonance in nanopillar structures pumped above bandgap with a femtosecond laser at relatively low fluences. Here, the pump‐induced refractive index modulation is ascribable to the photogeneration of free carriers. An even faster modulation speed (on the sub‐ps time scale) has been recently reported from GaP nanodisks^[^ ^22^ ^]^ by exploiting instantaneous optical Kerr effect and two‐photon absorption when pumping below the bandgap, even though at the expense of higher fluences of the control pulse. A comparison between the two nonlinear regimes of modulation (i.e., with the energy of the pump photon below or above the bandgap of the semiconductor) was conducted by some of the present authors for Si nanobrick metasurfaces.^[^ ^20^ ^]^

Although these results are very promising, research on ultrafast all‐dielectric nanoantennas and metasurfaces has been limited to the study of individual resonances over a relatively narrow spectral range, despite the fact that all‐dielectric nanostructures typically support multiple Mie resonances. The possibility of simultaneous ultrafast control of multiple resonances could be beneficial for a variety of applications, especially in planar configurations. As an example, multiresonant metasurfaces enable advanced functionalities, from full‐color computational imaging,^[^ ^23^ ^]^ to molecular barcoding,^[^ ^24^ ^]^ in addition to broadband light harvesting^[^ ^25^, ^26^ ^]^ and wide tunability.^[^ ^27^ ^]^ Moreover, a rather general aspect involved in the all‐optical modulation of dielectric nanoantennas under interband excitation has been so far overlooked: the spatial inhomogeneity with which electrons and holes are photogenerated by the control pulse, and the subsequent ultrafast dynamics following carrier diffusion at the nanoscale. Actually, Rudenko and coworkers have theoretically predicted how the inhomogeneity of free carrier generation can eventually induce an optical symmetry breaking in a silicon nanosphere.^[^ ^28^ ^]^ However, the effects of such spatio‐temporal transients on the all‐optical modulation from dielectric nanoantennas have not been experimentally investigated yet.

In this work, we perform an experimental and theoretical investigation on the ultrafast all‐optical response of multiresonant nanoantennas consisting of ${\text{Al}}_{.18}{\text{Ga}}_{.82}\text{As}$ nanopillars. Via broadband pump‐probe spectroscopy, we identify four distinct resonances and demonstrate geometrical tuning of the nonlinear resonant optical response by acting on the pillar radius. The ultrafast pump‐probe experiments are complemented by a semiclassical modeling of the transient reflectivity modulation, which takes into account the diffusion of the photogenerated free carriers inside the nanopillars as a key process governing their nonequilibrium optical response. This model enables us to reproduce and explain the full dynamics of the all‐optically modulated transient reflectivity, including the delayed rise time which is assigned to spatio‐temporal inhomogeneities, in the dynamics of photogenerated carriers, taking place at the nanoscale.

Our results offer an in‐depth insight into the physics of ultrafast modulation of dielectric nanostructures and pave the way to a refined approach in the design of flat‐optics ultrafast devices, including reconfigurable multiplexers, of interest for telecom and sensing applications.

## 2 Multiresonant Geometrically Tunable Nonlinear Optical Response

**Figure 1:**
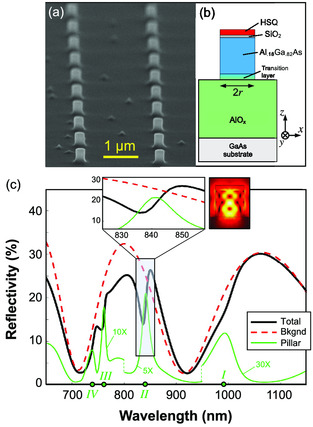
a) SEM image of the fabricated sample. b) Sketch of the nanopillar model. HSQ: hydrogen silsesquioxane. c) Simulated reflectivity (continuous black), substrate background reflectivity (dashed red), and reflectivity computed from the far fields scattered by the nanopillar only, magnified for better reading (solid green). Green points on the horizontal axis mark the spectral positions of the four nanopillar resonances in the considered wavelength range: $\lambda_{I}=993\;\text{nm}$, $\lambda_{\text{II}}=842\;\text{nm}$, $\lambda_{\text{III}}=761\;\text{nm}$, and $\lambda_{\text{IV}}=738\;\text{nm}$. Inset shows (left panel) a zoom‐in on resonance II highlighting the Fano‐like character of this resonance, and (right panel) the corresponding (normalized) electric field intensity pattern, exhibiting a magnetic quadrupolar character.

Among the many different platforms reported to date for all‐dielectric nanoantennas, those based on GaAs are particularly promising because of the direct bandgap of the material, enabling efficient excitation with visible light. Moreover, AlGaAs offers the possibility of a fine‐tuning of the bandgap by acting on the ratio between Al and Ga, which is an invaluable extra degree of freedom in view of further technological developments. For this reasons, the linear and nonlinear optical properties of GaAs and AlGaAs nanoantennas have been the subject of intensive research in the last few years.^[^ ^21^, ^29^, ^30^ ^]^ We thus selected a technological platform based on a 400 nm ${\text{Al}}_{.18}{\text{Ga}}_{.82}\text{As}$ nanopillar supported on an ${\text{AlO}}_{x}$/GaAs substrate (see Experimental Section for details). A scanning electron microscopy (SEM) image of the sample is shown in **Figure** **1**a. A 1 μm‐thick layer of ${\text{AlO}}_{x}$ isolates the AlGaAs pillars from the high‐index GaAs wafer, enabling the onset of high‐quality factor resonances. This configuration is thus expected to provide a highly structured reflectivity spectrum, arising from the interplay between two back‐scattering channels: resonant scattering from the nanopillars and a broadband background from the GaAs substrate.

To address the multiresonant design and tuning capability in reflection for this platform, we developed an electromagnetic model based on a finite‐element method using a commercial software (COMSOL multiphysics 5.4). Given the large distance between the meta‐atoms (2 μm in a square lattice configuration), which leads to a negligible near‐field interaction, we estimated the sample reflectivity from an individual nanopillar supported on an area corresponding to the unit cell of the array. This procedure enabled us to avoid spectral artifacts in the simulated reflectivity, such as sharp features arising in correspondence of the appearance/disappearance of diffracted orders. The incident beam is modeled as a monochromatic plane wave, with wavelength varying in the spectral range of interest, impinging at normal incidence.

We solved for the scattered field with a background field defined analytically according to Fresnel equations. Given the symmetry of the structure and of the background field, we simulated a quarter of a cylinder with perfect electric conductor (PEC) boundary conditions (BCs) in the *x,z* plane and perfect magnetic conductor (PMC) BCs in the *y,z* plane (Figure 1b). Perfectly matched layers (PMLs) have been added on the remaining boundaries. The reflectivity spectrum is then retrieved from the following formula

(1) $$R=\frac{1}{2P_{0}}\int_{\Sigma }\text{Re}(E\times H^{*})\cdot \hat{n}dA$$

where $P_{0}$ is the power of the impinging wave on the considered integration domain, Σ is a surface above the nanopillar corresponding to the numerical aperture (NA) of the experimental setup, which is equal to 0.2, **E** and **H** are the total electric and magnetic fields back‐reflected by the structure, and $\hat{n}$ is the outgoing normal unit vector perpendicular to Σ.

The simulated linear reflectivity at normal incidence for pillar radius r=243 nm is shown in Figure 1c (black line). The spectrum exhibits sharp asymmetric dips, superimposed to broader strong modulations which are roughly 200 nm wide. To explain such peculiar features in the calculated wavelength dependent reflectivity, we also calculated the reflection spectra arising only from the substrate (red dashed line in Figure 1c) or from the nanopillar (green lines in Figure 1c). When computing substrate reflectivity, the fields in Equation (1) are those corresponding to the background fields defined by Fresnel equations for the three media layer composed by GaAs bulk, the 1 μm‐thick ${\text{AlO}}_{x}$ spacer and air on top. When considering the contribution to reflectivity arising only from the nanopillar, the integrated fields are the far fields calculated from the scattered near fields of the nanopillar. According to such a disentanglement of the reflectivity contributions, we can ascribe the smoother and broader modulations to etalon effects inside the ${\text{AlO}}_{x}$ slab, whereas the sharp dips can be attributed to the onset of four distinct Mie resonances in the nanopillar (labeled with roman numbers in Figure 1c). Note that the total reflectivity (black curve in Figure 1c) exhibits asymmetric dips because of a Fano‐like mechanism due to the coherent interplay between the two back‐scattering channels, from the substrate and from the pillars. Consequently, the reflectivity is suppressed in the blue wing of the resonance and is, vice versa, increased on its red wing, as shown in the inset of Figure 1c. Due to the Fano resonance, the amplitude of the dips in the total reflectivity spectrum is significantly higher than the height of the peaks of the isolated nanopillars, showing that this mechanism is beneficial for obtaining strong resonant features. This is especially true for higher order resonances (II, III, IV in Figure 1c) that tend to exhibit a more pronounced subradiant behavior. These sharp features with a Fano‐like character in the optical response could be extremely promising in view of an effective all‐optical modulation of the platform.

**Figure 2:**
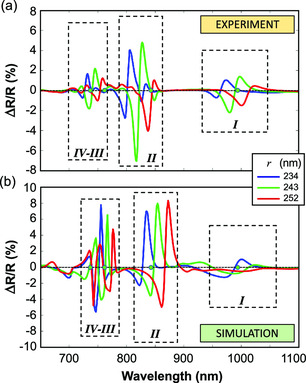
a) Experimental and b) simulated transient differential reflectivity at 2 ps time delay corresponding to the peak of the signal, for three AlGaAs nanopillars with radius r=234 nm (blue), r=243 nm (green), and r=252 nm (red). Green points on the horizontal axis correspond to the resonances of the r=243 nm pillar of Figure 1.

We investigated the ultrafast all‐optical modulation of all the four Mie resonances exhibited by AlGaAs nanoantennas in the 650–1150 nm spectral range, by broadband pump‐probe spectroscopy experiments. **Figure** **2**a shows the measured ΔR/R spectrum at the fixed time delay τ=2 ps, corresponding to the maximum of the transient signal, for three nanoantenna samples fabricated with different pillar radii r=234, 243, and 252 nm. Note that under a moderate pump fluence $F∼45\;\text{μJ}\;{{\text{cm}}^{-}}^{2}$, a sizable reflectivity modulation of several % is achievable around all the four resonances of the nanopillar, giving rise to both positive and negative variations with characteristic bandwidth comparable with the widths of the pillar resonances estimated from the numerical simulations of Figure 1c. By comparing the three ΔR/R spectra (obtained with the same pump fluence), we found a sizable red shift upon increasing the pillar radius *r*. From these spectra we estimated a tunability of the multiple resonances in the transient optical response of about ±30 nm under 10 nm increase/decrease in the pillar radius *r*. The experimental spectral shift can be indeed reproduced numerically, see Figure 2b, by varying the geometrical parameters of the pillar and including the effect of the photoexcitation, as explained in the following section.

## 3 Ultrafast Photoinduced Dynamics

**Figure 3:**
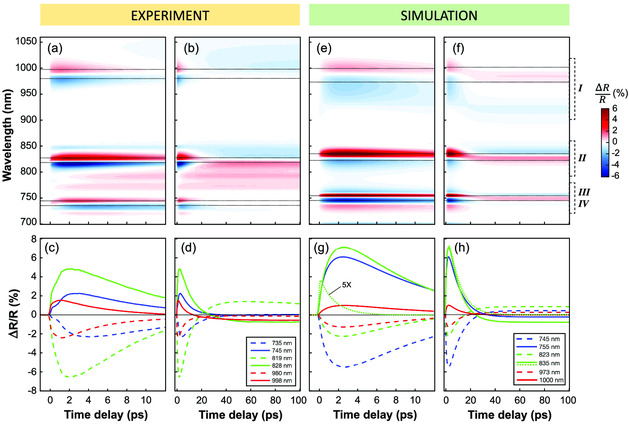
a,b) Experimental transient ΔR/R map recorded for the nanoantenna with pillar radius r=243 nm, on a) the short time scale and b) the long time scale. c,d) Experimental transient ΔR/R temporal traces at selected maxima (continuous lines) and minima (dashed lines) of the ΔR/R maps, corresponding to the dotted black lines in panels (a) and (b), respectively. e–h) Same as (a–d) for numerically simulated ΔR/R assuming a pillar radius r=234 nm. Panel (g) also shows the contribution to the dynamics at 835 nm (dotted green) arising from $N_{S}$ population only (5 times magnified for better reading). Panel (h) also shows the temporal trace retrieved by numerical simulation without considering thermo‐optical effects arising from lattice heating at 835 nm (dotted green): note the absence of sign change.

To gain a deeper understanding of the transient behavior induced in multiresonant nanoantennas by ultrafast photoexcitation, we recorded the ΔR/R spectra as a function of the pump‐probe time delay. A similar relaxation dynamics was observed for the three different nanoantenna configurations, including two different relaxation time scales: a fast one of about tens of picoseconds (ps) and a residual signal on the hundreds ps scale. We report the transient reflectivity ΔR/R(λ,τ) maps for the sample with r= 243 nm in **Figure** **3**a,b.

The dynamical response is analyzed in Figure 3c,d, by taking temporal cross sections of the ΔR/R(λ,t) maps at selected wavelengths, corresponding to the positive (solid lines) and negative (dashed lines) lobes observed around the three main resonances of the nanoantenna, namely, resonance I (red traces), resonance II (green traces), and resonance III (blue traces). First of all, as anticipated in the previous section, we note the absence of an instantaneous response in all the dynamical traces, regardless of the probe wavelength (Figure 3a,c). In fact, when the pump photon energy is above the gap (as in the present case), only linear absorption occurs and contributions from instantaneous two‐photon absorption, which manifest when pumping below the bandgap, are negligible at the used fluences, in agreement with previous studies.^[^ ^20^ ^]^ Most interestingly, we observe that the peak of the ΔR/R signal is achieved at a time delay (of about 2 ps) that is at least one order of magnitude larger than the pump‐pulse duration ( ≈150 fs). This indicates the presence of a delay mechanism in the transient optical response of all‐dielectric nanostructures that has been so far overlooked.

After the peak, the signal decays almost exponentially with a time constant of about 8 ps, and reaches a plateau on a time scale of 100 ps (Figure 3b). However, note that the amplitude of this plateau is strongly dispersed with the probe wavelength, and gives rise to a sign change for most of the signal traces (of Figure 3b,d), and in particular around resonance II (819 and 828 nm time traces).

**Figure 4:**
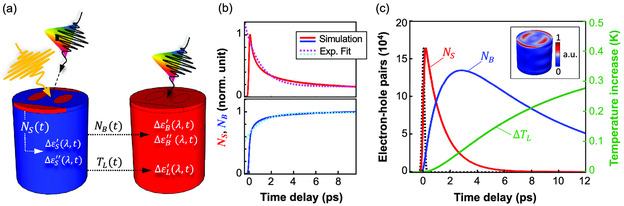
a) Sketch of the rationale of the inhomogeneous nonlinear model. A pump pulse (yellow arrow) impinges on the nanostructure, generating $N_{S}$ electron–hole pairs in hot spots localized at the top of the pillar (red spots on the left cylinder). These electron–hole pairs then diffuse in the bulk, giving rise to a population of $N_{B}$ electron–hole pairs distributed in the whole volume of the pillar (red cylinder on the right). The populations $N_{S}$ and $N_{B}$ preside over the permittivity variations $\Delta \varepsilon_{S}$, $\Delta \varepsilon_{B}$ that are monitored as a variation of the reflectivity of a broadband probe pulse (rainbow arrow) impinging on the nanopillar. Finally, nonradiative recombination provides lattice heating, detailed by the temperature $T_{L}$, which determines a variation $\Delta \varepsilon_{L}$. b) Identification of effective diffusion time constants on the basis of the 1D model of Equation (2): numerical solutions (normalized to the peak) from Equation (4) (solid red) and from Equation (5) (solid blue) neglecting electron–hole recombination are compared with single exponential dynamics (dotted curves) with the same time constant $\tau_{D}=1.3$ ps, fitted for t>0.5 ps. c) Dynamical variables $N_{S}$, $N_{B}$, and $T_{L}$ as a function of the pump‐probe delay, retrieved by numerical analysis of Equation ((6), (7))–(8), together with pump pulse intensity temporal profile (dashed black line). Inset shows the absorption pattern computed with full‐wave numerical simulations at the pump wavelength of 400 nm.

With the aim of providing a consistent interpretation of all the main features observed in the transient ΔR/R spectra, we developed a semiclassical theoretical model for the nonlinear optical response of the AlGaAs nanopillar. A sketch of the rationale behind the model is presented in **Figure** **4**a. As ${\text{Al}}_{.18}{\text{Ga}}_{.82}\text{As}$ is a direct semiconductor with a bandgap of $E_{g}=1.65\;\text{eV}$ (≈750 nm),^[^ ^31^ ^]^ a pump beam at $\lambda_{P}=400$ nm (3.2 eV) impinging onto the structure generates, via interband transitions, a population of free carriers (both electrons in the conduction band and holes in the valence band). Note that, describing the complex refractive index as n=n′+in″, the imaginary part of ${\text{Al}}_{.18}{\text{Ga}}_{.82}\text{As}$ refractive index at 400 nm is relatively high (n″=1.95 ^[^ ^31^ ^]^), and implies an optical skin depth as short as $\delta =\lambda_{P}/(4\pi n\prime \prime )≃16\;\text{nm}$, meaning that the pump laser beam only penetrates the top layer of the semiconductor. A refined numerical simulation confirms this hypothesis, and indicates that the photogenerated electron–hole pairs are located in hot spots close to the upper face of the pillar (see inset in Figure 4c). The free carriers are then expected to move out of these hot spots and diffuse into the whole bulk of the pillar via bipolar diffusion.

A fully consistent modeling of such inhomogeneous spatio‐temporal dynamics (coupled to the subsequent photoinduced permittivity changes as detailed below) would require a strong computational effort. Moreover, such a brute‐force approach offers little insight into the physical mechanisms underlying the measured quantities. For these reasons, we opted for an agile reduced model, capable of capturing the key phenomena associated with the highly inhomogeneous distribution of the photogenerated electron–hole pairs in the nanostructure, to evaluate the variation of the dielectric permittivity and its relaxation dynamics. As a first approximation, we assumed no transverse spatial dependence for both the absorption and the diffusion processes, so as to reduce our model to a 1D problem in a single spatial coordinate along the height of the nanopillars (*z*‐axis in our geometry).

In this framework, the equation for carrier generation and diffusion reads as follows

(2) $$\frac{\partial N(z,t)}{\partial t}=G(z,t)-\frac{N(z,t)}{\tau_{R}}+D\frac{\partial^{2}N(z,t)}{\partial z^{2}}$$

where N is the electron–hole density inside the nanopillar, $\tau_{R}$ is the carriers recombination time constant, and *D* = 18 cm^2^ s^−1^ is the bipolar diffusion coefficient of photoexcited carriers measured in GaAs at room temperature.^[^ ^32^ ^]^ We used the diffusion coefficient of GaAs to estimate the AlGaAs one, which is justified given the relatively low concentration of Al in our samples and the more impactful approximation involved in the 1D modeling. In the aforementioned equation, *G* is the driving term, describing the impulsive photoexcitation, modeled as

(3) $$G(z,t)=G_{0}\exp(-\frac{z}{\delta })\exp(-\frac{2t^{2}}{\tau_{p}^{2}})$$

where $G_{0}=Q_{A}(\lambda_{P})\lambda_{P}I_{0}/(hc\delta )=3\times 10^{33}\;{\text{cm}}^{-3}\;s^{-1}$ is the driving peak amplitude corresponding to the conditions of photoexcitation in our experiments, i.e., with $I_{0}≃2.5\;\text{GW}\;{\text{cm}}^{-2}$, $Q_{A}(\lambda_{P})$ is the nanopillar absorption efficiency evaluated at the pump wavelength (typical value for the pillar sizes under consideration is ≈2.1), *δ* = 16 nm is the skin depth of absorption inside the material, $\tau_{p}=127\;\text{fs}$ (corresponding to 150 fs full width at half maximum of the pump pulse, in agreement with experimental excitation conditions), *h* is the Planck constant, and *c* is the speed of light in vacuum. Equation (2) is solved with Neumann BCs for $t>t_{0}=-3\tau_{p}$, and the time‐ and space‐dependent carriers concentration N(z,t) is retrieved.

Then we define two populations of carriers, $N_{S}$, comprised in the skin depth, and $N_{B}$, distributed in the bulk of the pillar, that is

(4) $$N_{S}(t)=\pi r^{2}\int_{0}^{\delta }N(z,t)dz$$

(5) $$N_{B}(t)=\pi r^{2}\int_{\delta }^{H}N(z,t)dz$$

where *r* is the radius and *H* = 400 nm is the height of the nanopillars.

The dynamics of $N_{S}$ and $N_{B}$ in absence of recombination processes (solid curves in Figure 4b) can be approximated, after the initial transient due to photoexcitation, with two exponential curves, having the same time constant $\tau_{D}$ (dotted curves in Figure 4b). A fitting procedure for time delay t>0.5 ps retrieves $\tau_{D}=1.3\;\text{ps}$. In this way, we achieved a further simplification in our model, as a single time constant governs over both the decay of $N_{S}$ and the increase in $N_{B}$. Following diffusion, once the carriers are delocalized in the nanopillar, recombination takes place. In agreement with reference,^[^ ^21^ ^]^ we assumed surface trap‐assisted nonradiative recombination as the dominant process (disregarding higher order bimolecular and Auger recombination considering the relatively small fluences of our experiments), with a time constant $\tau_{R}=8\;\text{ps}$ estimated on the basis of the decay dynamics observed in the experiments. Note that nonradiative recombination implies generation of phonons in the lattice and subsequent increase in the lattice temperature $T_{L}$ of the nanopillar. Also, in principle, carriers recombination can occur during the diffusion process as well. However, the observation that the time constant for recombination ($\tau_{R}$) is much larger than the one for diffusion ($\tau_{D}$) enables one to separate the temporal scales of the two processes. The temporal dynamics of $N_{S}$, $N_{B}$, and $T_{L}$ variables can thus be modeled according to a system of three ordinary differential equations, detailed in the Experimental Section as Equation ((6), (7))–(8). Their solution under excitation conditions corresponding to the ones used in our experiments is shown in Figure 4c.

Each of the two populations of charge carriers corresponds to a plasma density $n_{S,B}=N_{S,B}/V_{S,B}$, where $V_{S}$ and $V_{B}$ are the volumes of the hot spots and of the whole bulk of the nanopillar, respectively. These transient plasmas are, in turn, responsible for a transient change in the AlGaAs permittivity, $\Delta \varepsilon_{S,B}(\lambda ,t)$, according to two distinct processes: 1) the free carrier Drude mechanism and 2) the interband transition modulation due to band filling. The lattice heating also contributes with a permittivity change $\Delta \varepsilon_{L}(\lambda ,t)$ due to the thermo‐optical effect (see Experimental Section for further details). The total permittivity variation is therefore defined as the sum of all these contributions, namely, $\Delta \varepsilon (\lambda ,t)=\Delta \varepsilon_{S}(\lambda ,t)+\Delta \varepsilon_{B}(\lambda ,t)+\Delta \varepsilon_{L}(\lambda ,t)$. Finally, the evaluation of the transient optical response is obtained by assuming a perturbative regime of the reflectivity with respect to the permittivity variation (see Experimental Section).

With this model we first performed numerical simulations aimed at corroborating the multiresonant tunability of the nonlinear optical response observed in the experiments of Figure 2a. The simulated ΔR/R spectra at 2 ps time delay for pillars with three different radii, namely, r=234, 243, and 252 nm, are shown in Figure 2b. Note that the model is capable of reproducing all the main features observed in the experiments (cf. Figure 2a), apart from a systematic red shift of about 10 nm, i.e., the theoretical spectrum retrieved for the nanopillar with r=234 nm (blue curve in Figure 2b) well matches the experimental spectrum recorded for the nanopillar with r=243 nm (green curve in Figure 2a). The numerical simulations also tend to overestimate the amplitude of the transient reflectivity modulation by about a factor of 2, considering that the simulation results of Figure 2b have been obtained assuming an incident pump fluence of $25\;\text{μJ}\;{\text{cm}}^{-2}$, against the $45\;\text{μJ}\;{\text{cm}}^{-2}$ used in the experiments.

Figure 3e shows the simulated ΔR/R map (for r=234 nm) on the short time scale, and the temporal cross sections of the simulated map at different probe wavelengths, corresponding to selected maxima (solid lines) and minima (dashed lines) of the map, are shown in Figure 3g. A comparison with the experimental results of Figure 3a,c (for r=243 nm) indicates that the model is capable of reproducing the main features of transient reflectivity dynamics. Note that, if the contribution arising from the second population of carriers $N_{B}$ is neglected, the model prediction (dotted green line in Figure 3g) dramatically deviates from the measurements (cf. solid green line in Figure 3c), both in terms of the intensity of the modulation and, most importantly, in terms of its dynamical behavior. The reduced model for electron–hole diffusion based on the two populations $N_{S}$ and $N_{B}$, thus explains the 2 ps delay in the onset of the signal peak, even though in some traces (e.g., blue and red traces) it becomes apparent that more than two time constants are necessary to satisfactorily reproduce the experiments.

The simulated ΔR/R map on a longer time scale and the corresponding ΔR/R dynamics at selected wavelengths (again computed for r=234 nm) are shown in Figure 3f,h, respectively. Note that the model also correctly retrieves the sign change observed in the experimental data of Figure 3b,d, especially around resonances II and III. This behavior is ascribable to the thermo‐optical effect following lattice heating, which typically provides an opposite contribution to the modulation of the optical response compared with the one arising from the Drude mechanism triggered by electron–hole pairs formation.^[^ ^20^ ^]^ Interestingly, when disregarding thermo‐optics (dotted green trace in Figure 3h), no sign change is observed in the simulations (compared with solid green trace in Figure 3h). Good agreement with the experimental data is found upon considering the following values for the thermo‐optical coefficients $\eta_{1}=15\times 10^{-4}\;K^{-1}$ and $\kappa =400\;K^{-1}\;{\text{cm}}^{-1}$, which turned out to be significantly higher than the typical values reported in the literature. For example, $\eta_{1}=3.2\times 10^{-4}\;K^{-1}$ was measured in GaAs at 1 μm wavelength.^[^ ^33^ ^]^ However, it is worth saying that on the ultrabroad spectral range of our experiments, the thermo‐optical coefficients are expected to be strongly dispersed with wavelength, dramatically increasing with the photon energy, especially when approaching the bandedge (see, e.g., Harris et al.^[^ ^34^ ^]^ and references therein). The value retrieved from our fitting can be thus considered as a rough estimation of the average thermo‐optical coefficient on the whole band of interest.

## 4 Conclusion and Outlook

The transient optical response of multiresonant all‐dielectric nanoantennas following the excitation with intense ultrashort laser pulses has been reported. The simultaneous all‐optical control over four distinct resonances of ${\text{Al}}_{.18}{\text{Ga}}_{.82}\text{As}$ nanopillars is experimentally demonstrated via broadband pump‐probe spectroscopy. Similar to what is observed in the linear regime, the transient optical response strongly depends on the nanopillar radius. A semiclassical nonlinear optics model based on a three‐rate‐equation model description of the internal dynamics of the photoexcited nanoantennas, combined with full‐wave electromagnetic simulations, is capable of reproducing all the main spectral and temporal features observed in the experiments. The Drude plasma (intraband) response and the Pauli blocking of interband transitions, following pump‐induced band filling, turned out to be the dominant processes presiding over the picosecond time‐scale dynamics, with the latter being mostly relevant in the high energy range of the considered spectrum. Conversely, the thermo‐optical effect induced by lattice heating after nonradiative electron–hole recombination presides over the response on the few hundred picosecond time scale. We also point out the key role played by bipolar diffusion of the photogenerated electron–hole pairs during the very early stage of the dynamics. On the basis of the remarkable agreement between experiments and simulations, we believe that our model can provide a support for the design of ultrafast all‐optically reconfigurable multiresonant nanoantennas and metasurfaces. In particular, when dealing with all‐optical signal processing in modern wavelength division multiplexing (WDM) optical networks, the extraction/insertion of a single channel from time‐interleaved optical signals is a key functionality, demanding for very high modulation speed (10–100 Gb s^−1^) at selected operation wavelengths.^[^ ^35^, ^36^ ^]^ We found that all‐dielectric nanoantennas based on AlGaAs on ${\text{AlO}}_{x}$ enable a relatively high contrast reflectivity modulation (up to 7% in modulus) with moderate pump fluence ($\approx 45\;\text{μJ}\;{\text{cm}}^{-2}$) due to the onset of high‐quality factor resonances of Fano type, which can be easily tuned by geometrical means. Our results can thus be of interest for the development of ultrafast all‐optically reconfigurable filters for add/drop multiplexing in unprecedented ultracompact (flat‐optics) configurations.

## 5 Experimental Section

5.1

5.1.1

##### Nanostructures Fabrication

The active layer was grown by molecular beam epitaxy on top of a 1 μm‐thick ${\text{Al}}_{.98}{\text{Ga}}_{.02}$ layer. In addition, two 90 nm‐thick layers with linearly varying Al molar fraction were grown at the two sides of the aluminum‐rich substrate to improve its adhesion with the adjacent crystalline layers. Spin coating of a 100 nm‐thick layer of high‐resolution negative resist hydrogen silsesquioxane (HSQ) was preceded by the plasma‐enhanced chemical vapor deposition of ${\text{SiO}}_{2}$ (10 nm), to improve HSQ adhesion, and followed by electron‐beam lithography at 20 kV. Thus, the pattern was transferred in the active layer via inductively coupled plasma‐reactive‐ion etching, at 35 W plasma power, where the etching depth of 400 nm was controlled by a laser interferometer. Finally, the aluminum‐rich substrate revealed by dry etching was oxidized at 390 °C for 30 min, under a precisely controlled water vapor flow with $N_{2}$:$H_{2}$ gas carrier. For more details on the fabrication, see the study by Carletti et al.^[^ ^29^ ^]^

##### Ultrafast Spectroscopy

The setup is based on an amplified Ti:sapphire laser (Coherent, Libra) producing 150 fs pulses at 800 nm wavelength. We chose a nondegenerate configuration with the pump pulse tuned at 400 nm, obtained by second harmonic generation of the laser fundamental, to be outside of the probed spectral range and well above the bandgap of AlGaAs to photoexcite it via interband absorption. To simultaneously monitor all the four expected resonances, we used a probe pulse extending from 400 to 1100 nm obtained by white light continuum generation in a 2 mm‐thick sapphire plate. The pump and the probe pulses were focused on the sample at normal incidence and the reflected probe light was dispersed in an optical multichannel analyzer (OMA). To obtain the variation of the sample reflectivity induced by the photoexcitation, we measured the differential reflectivity $\Delta R/R=R_{\text{ON}}/R_{\text{OFF}}-1$, where ΔR is the difference between the reflected probe spectrum in the presence, $R_{\text{ON}}$, and in the absence, $R_{\text{OFF}}$, of the pump pulse. The ΔR/R signal was resolved as a function of the probe wavelength *λ* and time delay *τ*, varied with an optical delay line, with an overall temporal resolution of ≈150 fs.

##### Numerical Modeling of Nonequilibrium Dynamics and Transient Optical Response

The three rate equations governing the evolution of $N_{S}$, $N_{B}$, and $T_{L}$ read as follows

(6) $$\frac{dN_{S}}{dt}=\Phi_{P}(t)-\frac{N_{S}}{\tau_{D}}$$

(7) $$\frac{dN_{B}}{dt}=\frac{N_{S}}{\tau_{D}}-\frac{N_{B}}{\tau_{R}}$$

(8) $$\frac{dT_{L}}{dt}=\frac{h\nu_{P}}{C_{L}V}\frac{N_{B}}{\tau_{R}}$$

where $\nu_{P}=c/\lambda_{P}=749$ THz is the pump beam central frequency (*c* being the speed of light in vacuum), $C_{L}=1.86\times 10^{6}\;{\text{Jm}}^{-3}K^{-1}$ is the specific heat of AlGaAs, and *V* is the nanopillar volume. In aforementioned equations, we have disregarded the cooling dynamics of the lattice, taking place on a time scale much longer than that considered in the current study, and $\Phi_{P}$ is the absorption rate of the pump beam, modeled with the following equation

(9) $$\Phi_{P}(t)=\sqrt{\frac{2}{\pi }}\frac{Q_{A}(\lambda_{p})\pi r^{2}F\lambda_{P}}{\tau_{p}hc}\exp(-\frac{2t^{2}}{\tau_{p}^{2}}).$$

Each of plasma densities $n_{S}$ and $n_{B}$ are responsible for a modulation of the AlGaAs permittivity in the corresponding volume, $\Delta \varepsilon_{S,B}(\lambda ,t)=\text{Re}\{\Delta \varepsilon_{S,B}^{\text{Drude}}(\lambda ,t)\}+i\text{Im}\{\Delta \varepsilon_{S,B}^{\text{Drude}}(\lambda ,t)$}, where the individual terms are defined by the Drude permittivity model^[^ ^37^ ^]^

(10) $$\text{Re}\{\Delta \varepsilon_{S,B}^{\text{Drude}}(\lambda ,t)\}=-[\frac{1}{m_{e}}+(\frac{m_{hh}^{\frac{1}{2}}+m_{lh}^{\frac{1}{2}}}{m_{hh}^{\frac{3}{2}}+m_{lh}^{\frac{3}{2}}})]\frac{n_{S,B}e^{2}}{\varepsilon_{0}\left[{\left(\frac{2\pi c}{\lambda }\right)}^{2}+\Gamma^{2}\right]},$$

(11) $$\text{Im}\{\Delta \varepsilon_{S,B}^{\text{Drude}}(\lambda ,t)\}=-\text{Re}\{\Delta \varepsilon_{S,B}^{\text{Drude}}(\lambda ,t)\}\frac{\lambda \Gamma }{2\pi c}$$

In the aforementioned formulas, $m_{e}=0.084\;m_{0}$, $m_{lh}=0.099\;m_{0}$, and $m_{hh}=0.573\;m_{0}$ are the effective electron, light hole, or heavy hole masses in ${\text{Al}}_{.18}{\text{Ga}}_{.82}\text{As}$ derived from the study by Adachi,^[^ ^31^ ^]^ $m_{0}$ is the free electron mass, *e* is the elementary charge, $\varepsilon_{0}$ is the vacuum permittivity, and $\Gamma =13.7\times 10^{12}\;\text{rad}\;s^{-1}$ is the Drude broadening estimated from carriers mobility.^[^ ^38^ ^]^

As the conduction band starts being filled with electrons promoted from the valence band by photoexcitation, the same transition becomes forbidden because of Pauli exclusion principle, causing a modulation of the absorption coefficient Δα(λ,t), or, equivalently, of the imaginary part of AlGaAs refractive index, Δn″(λ,t)=λΔα(λ,t)/(4π). The latter is calculated in agreement with the Bennett's seminal paper,^[^ ^39^ ^]^ using AlGaAs parameters from the literature,^[^ ^31^ ^]^ even though the energy gap $E_{g}$ is treated as a fitting parameter to properly account for the strong simplification introduced by the parabolic band approximation. We took $E_{g}=1.75\;\text{eV}$, against the nominal value of 1.65 eV typically assumed for ${\text{Al}}_{.18}{\text{Ga}}_{.82}\text{As}$. Then, the corresponding modulation of the real part of the refractive index, Δn′(λ,t), is retrieved by Kramers–Kronig analysis, and the complex permittivity variation $\Delta \varepsilon_{S,B}^{\text{Pauli}}(\lambda ,t)=\text{Re}\{\Delta \varepsilon_{S,B}^{\text{Pauli}}(\lambda ,t)\}+i\text{Im}\{\Delta \varepsilon_{S,B}^{\text{Pauli}}(\lambda ,t)\}$ is finally obtained as $\text{Re}\{\Delta \varepsilon_{S,B}^{\text{Pauli}}\}=2[n\prime \Delta n\prime -n\prime \prime \Delta n\prime \prime ]$ and $\text{Im}\{\Delta \varepsilon_{S,B}^{\text{Pauli}}\}=2[n\prime \Delta n\prime \prime +n\prime \prime \Delta n\prime ]$.

The residual transient signal, observed on the 100 ps time scale, can be understood considering the change in the refractive index due to the temperature increase in the AlGaAs lattice, which takes place because of thermo‐optical effects, giving rise to modulation of permittivity $\Delta \varepsilon_{L}=\text{Re}\{\Delta \varepsilon_{L}(\lambda ,t)\}+i\text{Im}\{\Delta \varepsilon_{L}(\lambda ,t)\}$, where

(12) $$\text{Re}\{\Delta \varepsilon_{L}(\lambda ,t)\}=2[n\prime (\lambda )\eta_{1}-n\prime \prime (\lambda )\frac{\lambda }{4\pi }\kappa ]\Delta T_{L}(t)$$

(13) $$\text{Im}\{\Delta \varepsilon_{L}(\lambda ,t)\}=2[n\prime \prime (\lambda )\eta_{1}+n\prime (\lambda )\frac{\lambda }{4\pi }\kappa ]\Delta T_{L}(t)$$

In the aforementioned equations, $\eta_{1}$ and *κ* are the real and imaginary thermo‐optical coefficients of AlGaAs, respectively, whose values are fitted on the experimental data, and $\Delta T_{L}(t)=T_{L}(t)-T_{\text{env}}$ is the temperature increase in the semiconductor with respect to the environment temperature $T_{\text{env}}$.

By considering a perturbative regime, with respect to permittivity variation, we finally calculated the transient reflectivity dynamics as

(14) $$\frac{\Delta R}{R}(\lambda ,t)=c_{1}(\lambda )\Delta \varepsilon \prime (\lambda ,t)+c_{2}(\lambda )\Delta \varepsilon \prime \prime (\lambda ,t)$$

where Δε′(λ,t) and Δε″(λ,t) are the aforementioned real and imaginary permittivity changes induced by the pump beam, $c_{1}(\lambda )$ and $c_{2}(\lambda )$ are the reflectivity spectral coefficients, meant to approximate the partial derivatives ∂R/∂ε′ and ∂R/∂ε″. These have been numerically evaluated as

(15) $$c_{j}(\lambda )=\frac{R\left[\varepsilon (\lambda )+{\bar{\Delta \varepsilon }}_{j}\right]-R\left[\varepsilon (\lambda )\right]}{\left|{\bar{\Delta \varepsilon }}_{j}\right|},\;\;\;\;j=1,2$$

where ε(λ) is the complex permittivity of ${\text{Al}}_{.18}{\text{Ga}}_{.82}\text{As}$ and ${\bar{\Delta \varepsilon }}_{j}$ is a uniform small perturbation. In particular, in our simulations we assumed a perturbation of amplitude 0.1 for calculating $c_{1}(\lambda )$, and *i*0.1 for calculating $c_{2}(\lambda )$. Due to the perturbative approach, the arbitrary choice of the modulus of the perturbation does not influence the resulting reflectivity spectral coefficient, as long as the perturbation amplitude is in the range of |Δε(λ,t)|. Finally, note that the linear dependence on spectral coefficients $c_{j}(\lambda )$ in Equation (14) implies that the specific quality factor of nanoantenna resonances is influencing the amplitude of transient reflectivity variation and the spectral width of its lobes, i.e., the size of the resonances’ shift, without affecting the transient reflectivity dynamics.

## Conflict of Interest

The authors declare no conflict of interest.

## Data Availability Statement

Research data are not shared.
